# Association Between Plasma Vitamin D2 and Type 2 Diabetes Mellitus

**DOI:** 10.3389/fendo.2022.897316

**Published:** 2022-06-01

**Authors:** Jing-Wan Qi, Bing Huang, Shuang Wang, Dan Song, Jing Xu, Ying Cui, Bin Guo

**Affiliations:** ^1^ Department of Microbiology and Biochemical Pharmacy, Pharmaceutical Sciences School of Jinzhou Medical University, Jinzhou, China; ^2^ Research Department, Dalian Innovation Center of Laboratory Medicine Mass Spectrometry Technology, Dalian, China; ^3^ Research Department, Clinical Mass Spectrometry Profession Technology Innovation Center of Liaoning Province, Jinzhou, China

**Keywords:** plasma VD2, type 2 diabetes, metabolism, China, relationship

## Abstract

**Objective:**

To investigate the relationship between plasma vitamin D2(VD2) and type 2 diabetes(T2DM).

**Method:**

Data from electronic medical records of 797 inpatients treated at Sun Yat Sen Memorial Hospital, Sun Yat-sen University between June 24, 2019 and December 24, 2020 were collected, and a total of 596 patients were enrolled after screening based on inclusion and exclusion criteria. Patients were divided into diabetic and non-diabetic groups according to whether they had T2DM. The Wilcoxon rank sum test was finally selected for the analysis of differences between groups according to the distribution of patients’ plasma VD2, and logistic regression models were used to find the corresponding influencing factors.

**Result:**

Of the 596 hospitalized patients, 138 (23.15%) were diagnosed with T2DM. The Wilcoxon test showed no statistically significant difference in plasma VD2 concentrations between the T2DM and non-T2DM groups (p=0.833). After adjustment for confounders by multivariate logistic regression, there was still no significant difference in plasma VD2 concentrations between the two groups (P=0.316, OR: 1.15 (0.88,1.49)). The uncorrelated relationship between VD2 and T2DM was not found to change after incorporating 12 indicators, including demographic characteristics, laboratory indicators and complications, into the logistic regression model by 3 steps, even the OR (1.08 (0.92,1.26)) did not change in the 3 models. Similarly, the adjusted ORs agreed that there was no statistical association between VD2 and T2DM.

**Conclusion:**

VD2 levels are similar in patients with T2DM compared to those without T2DM. Clinical caution should be exercised in giving VD2 supplementation to patients with T2DM unless other diseases requiring VD2 supplementation (e.g., rickets, osteoporosis) are present.

## Introduction

Diabetes is a group of serious metabolic diseases characterized by persistent hyperglycemia, the prevalence of diabetes has increased rapidly worldwide in recent years, with an estimated 451 million people living with diabetes worldwide in 2017 and this number expected to increase to 693 million by 2045 (1),causing great harm to the health of people all over the world (2), and diabetes is one of the important causes of long-term ill health and premature death, even more dangerous than AIDS (3).

In worldwide, 90% of people with diabetes are type 2 diabetics, of which insulin resistance is the main cause.Although the treatment of T2DM has improved over the last few decades, the factors that influence the development and progression of T2DM and its complications are numerous and complex, and remain poorly studied, making it essential to find innovative ways such as biomarkers or efficient treatment to prevent and manage the disease (1). At present, the latest progress in the treatment of T2DM focuses on drugs that enhance insulin secretion, sensitize and reduce the production of liver sugar (3). Meanwhile, studies (4–7) suggest that biomarkers may have predictive and diagnostic potential, also in the case of diabetes (6, 7). According to the TCM view of treating the disease before it happens, perhaps early prevention by finding biomarkers would be more meaningful. Metabolomics is a research approach that follows the ideas of genomics and proteomics to quantify all metabolites in an organism and to find the relative relationship between metabolites and physiopathological changes. Due to the continuous development of advanced analytical techniques and bioinformatics, metabolomics, an emerging approach in the field of systems biology, the analysis of metabolites in cells and tissues is often used as a tool for biomarker discovery (8–12).

It is not uncommon to find biomarkers related to diabetes and its complications by metabolomic approaches, and the association between indicators such as branched-chain amino acids, complex amino acids and carnitine and diabetes has been found (13–16). We believe that there are numerous metabolomic indicators about T2DM, and it is probable that there are still undiscovered indicators that deserve further exploration. In the process of reviewing the literature, we found previous studies have shown that the concentration of plasma VD (a fat-soluble vitamin) in diabetic patients is lower than that in non-diabetic patients (17, 18), that is VD deficiency can lead to increased of T2DM (19–21). In addition, assessment of VD status by plasma 25-hydroxy VD levels (25(OH)D) has been shown to be inversely associated with insulin resistance, but the mechanism of this association remains unclear. Another studies have shown that VD plays an important role in improving the risk of T2DM, which may be mediated by the effects of VD on beta cell function, insulin sensitivity, and systemic inflammation (22, 23). The relationship between low VD status and the prevalence of T2DM is generally consistent with the results of national and international studies, but there are no uniform findings regarding the correlation between VD and metabolic indicators in patients with T2DM.

More importantly, there are different types of VD, mainly VD2 and VD3. In previous studies, most of them were cohort studies with VD supplementation as an intervening factor, while fewer studies were conducted specifically on the association between plasma VD2 or VD3 and diabetes. It is known from a review that plasma VD2 can be used in screening for type 1 diabetes by specific methods (24), however, its association with T2DM is unclear. On the basis of the studies of VD2 and type 1 diabetes, we chose VD2 to study its association with type 2 diabetes in order to complement the existing studies. Further it can be used for whether VD2 is needed as a supplement for the prevention and treatment of patients with T2DM. We therefore designed this cross-sectional study: an attempt to explore whether there is an association between plasma VD2 concentration and T2DM,

## Materials And Method

### Research Design and Study Patients

**Figure 1:**
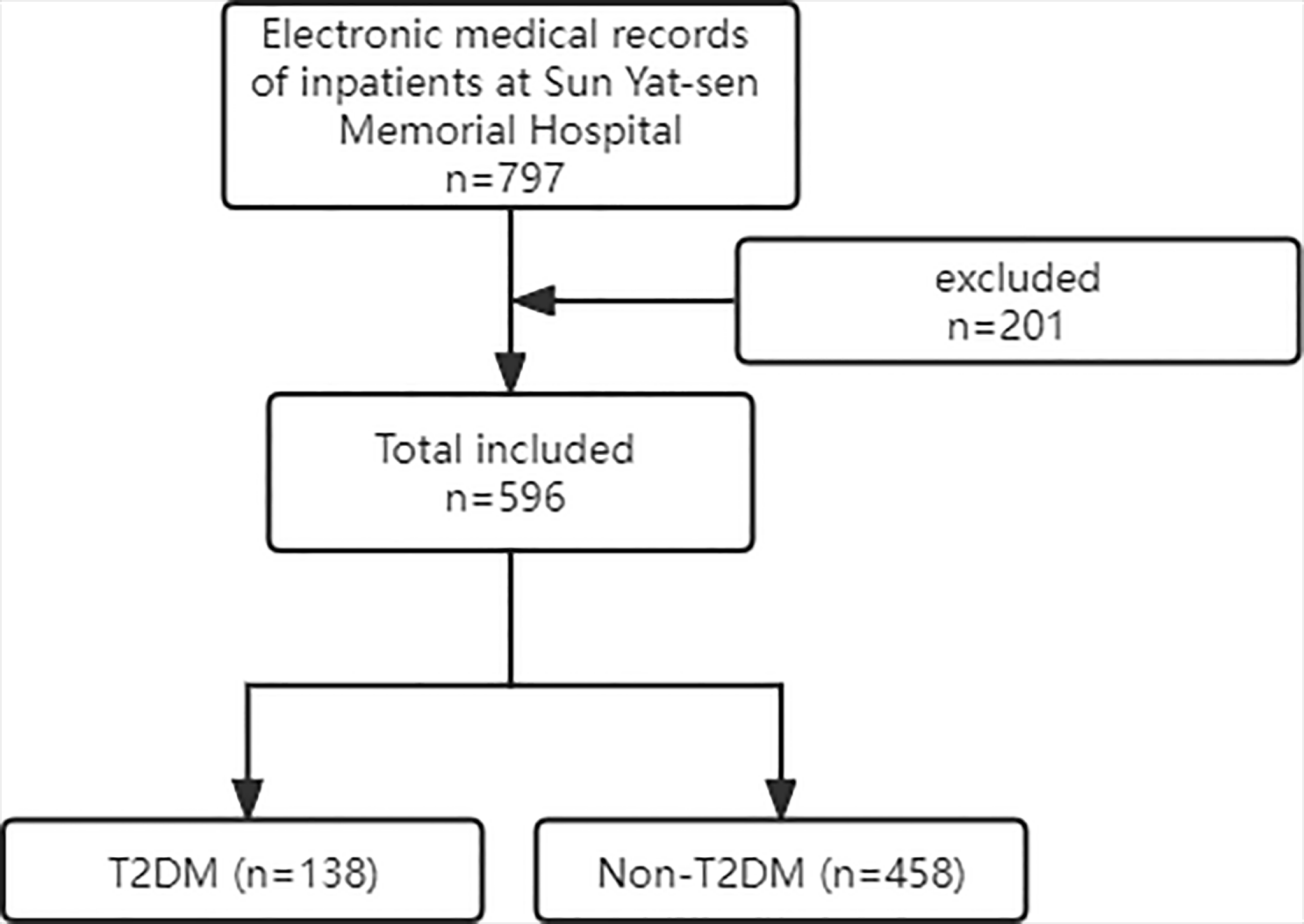
Article Flow Chart.

We collected electronic medical records of 797 inpatients at Sun Yat-sen Memorial Hospital, Sun Yat-sen University between June 24, 2019 and December 24, 2020. Inclusion criteria: 1) All selected candidates must have complete electronic medical records for the index of study interest; 2) 18 years old and above; 3) All enrollees were able to eat and drink normally, were not in the acute phase of disease or with serious life-threatening illness, and do not come from an ICU unit; 4) All selected candidates do not come from hospitalized persons during pregnancy or puerperium. Electronic medical records that do not meet the above requirements will be deleted. At last, 201 people whose medical records lacked information such as height, weight and blood pressure were excluded and a total of 596 subjects were enrolled (**Figure 1**). T2DM group should be diagnosed as T2DM according to the 1999 World Health Organization standard (25). The ethics Committee of Dalian R&S Kangtai Medical Testing Laboratory Co. authorized the ethics of this study and approved a waiver of informed consent due to the nature of the study. This is consistent with the Declaration of Helsinki.

### Collection and Definition

Data included demographic characteristics (Age, gender, BMI, smoking, drinking), Laboratory indicators (SBP, DBP, HDL-C, LDL-C, total cholesterol (CHOL), VD2) and complications (coronary heart disease (CHD), heart failure, diabetic nephropathy (DN), diabetic retinopathy (DR) and dyslipidemia). Indicators like age, gender, smoking and alcohol consumption/duration would require the physician’s nurse to ask and make additions, and patients who had smoked or consumed alcohol for more than 5 days a week were considered to have smoked or consumed alcohol. Other laboratory indicators are obtained by routine hospital tests. The subjects’ indicators were measured using standardized methods by trained physicians and nurses. Participants were asked to wear light weight clothing and remove their shoes while their height and weight were measured.

Age is calculated based on the date of birth and the number of years between the patient’s health check-up. Height and weight are each accurate to a decimal point. BMI was calculated by the ratio of body weight (kg) to height square (m), and BMI was classified into underweight (18.5 kg/m2), normal weight (18.5-23.9 kg/m2), obese (24-27.9 kg/m2) and overweight (≥28 kg/m2) according to Chinese adult standards (26).

To measure blood pressure, participants were asked to sit for 10 minutes before the measurement and have their right arm measured with a standard mercury sphygmomanometer. Diabetic nephropathy (DN) is diagnosed according to “An updated overview of diabetic nephropathy: Diagnosis, prognosis, treatment goals and latest guidelines” (27); Diabetic retinopathy (DR) was examined by our ophthalmologist with fundoscopy and, if necessary, fundus fluorescence angiography were used as the final differential diagnosis (28). The confirmation of CHD is done by coronary angiography (29) which is a gold standard; Heart failure diagnosis based on the” 2016 European Society of Cardiology Guidelines for the Diagnosis and Treatment of Acute and Chronic Heart Failure” (30). And the diagnosis of dyslipidaemias is done according to the “2016 ESC/EAS Guidelines for the Management of Dyslipidaemias” (31).We do not differentiate between patients’ smoking and drinking duration, nor do we grade the duration and severity of their diabetes and its complications. Nephropathy and retinopathy in non-T2DM are considered common nephropathy and retinopathy.

### Determination of VD2 by LC-MS/MS

The plasma VD2 concentrations were detected by a LC-MS/MS method, using an AB SCIEX TRIPLE QUAD™ 4500MD LC-MS/MS System (ABsciex, Toronto, Canada). Briefly, 10 μL of internal standard was added to 100 μL of plasma samples and then mixed with 300 μL of extraction solution. The supernatant was collected after vortex and centrifugation, subsequently injected for LC-MS/MS analysis. Calibrators and quality controls were prepared based on the same procedure.

Chromatographic analysis was performed on the Jasper™ HPLC system equipped with a Gemini 3 μm C18 column. Mobile phase A consisted of water with 0.1% formic acid, and mobile phase B consisted of acetonitrile with 0.1% formic acid. Thirty microliters of the sample solutions were injected into the LC system using a column temperature of 40°C and a flow rate of 0.8 mL/min. Mass spectrometry analysis was performed by multiple reaction monitoring in positive electrospray ionization mode. Optimized parameters for mass detection were as follows: curtain gas was 30 psi; collision gas was 6 psi; the temperature was 400°C; ion spray voltage was 5,500 V; ion source gas 1 was set at 30 psi; the duration was 5 min. All data were acquired and processed with Analyst Software, version 1.6.3 (Applied Biosystems).

### Statistical Analysis

The quantitative data subject to normal distribution were represented by mean and standard deviation (SD), and the T-test of two independent samples was used to determine whether the difference between the two groups was statistically significant. The quantitative data that did not follow the normal distribution were represented by median (interquartile range), and wilcoxon test was used to determine whether the difference between the two groups was statistically significant. Qualitative data were expressed by N (percentages), and chi-square test (or Fisher’s exact test where appropriate) was used to determine whether the differences between groups were statistically significant. Multivariate logistic regression was used to adjust the basic population information and possible confounding factors with statistically significant differences between the two groups, and the adjusted P value, odds ratio and 95% CI were obtained.

In logistic regression, different adjustment models was used to adjust for different confounding factors. First, we obtain an unadjusted OR value. Second, we use different models to adjust different confounding factors to obtain the corresponding OR value. In model 1, age, BMI (quantitative BMI data were introduced here) and smoking were adjusted. In model 2, age, BMI, smoking, SBP, HDL-C, LDL-C and CHOL were adjusted. Model 3 adjusted for age, BMI, smoking, SBP, HDL-C, LDL-C, CHOL and CHD.

R version 4.1.1 was used in statistic analysis. A two-tail p <0.05 means difference was statistically significant.

## Result

A total of 596 subjects were enrolled in this study, and they were divided into T2DM group (n=138) and non-T2DM group (n=458). As is shown in **Table 1**, the age of the patients in the diabetic group was 61.00 (55.00, 68.00) and it was significantly(p=0.002) higher than in the non-diabetic group 58.00 (49.00, 65.00). The gender distribution of patients in the diabetic group was not statistically different from the non-diabetic group (p=0.056). Patients in the diabetic group had a higher BMI than non-diabetic patients, when included as a continuous variable(p<0.001). When BMI was the categorical variable, a significantly higher proportion of diabetic patients were in the BMI>28 group (24(17.39%) VS 34(7.42%)). When discussing smoking and alcohol consumption, we found a higher percentage of patients in the diabetic group smoked [32(23.19%) VS 64(13.97%), p=0.014], while alcohol consumption was not statistically different from the non-diabetic group [28(20.29%) VS 73(15.94%), p=0.287].

**Table 1:** Clinical and biochemical characteristics of participants according to the occurrence of T2DM.

Variables	T2DM (138)	Non-T2DM (458)	*P*-value
Age (years)	61.00 (55.00, 68.00)	58.00 (49.00, 65.00)	0.002
Gender (Male)	74 (53.62%)	201 (43.89%)	0.056
BMI (kg/m2)	25.99 ± 3.09	24.36 ± 3.43	<0.001
BMI<18.5	1 (0.72%)	14 (3.06%)	<0.001
BMI≥18.5 and<24	24 (17.39%)	113 (24.67%)	
BMI≥24 and<28	89 (64.49%)	297 (64.85%)	
BMI≥28	24 (17.39%)	34 (7.42%)	
Smoking	32 (23.19%)	64 (13.97%)	0.014
Drinking	28 (20.29%)	73 (15.94%)	0.287
SBP (mmHg)	131.81 ± 18.11	127.92 ± 17.83	0.004
DBP (mmHg)	82.54 ± 9.42	81.50 ± 10.81	0.091
HDL-C (mmol/L)	1.21 (0.99, 1.44)	1.33 (1.14, 1.60)	<0.001
LDL-C (mmol/L)	2.75 (2.18, 3.33)	2.98 (2.38, 3.52)	0.022
CHOL (mmol/L)	4.90 (4.19, 5.69)	5.10 (4.50, 5.95)	0.047
VD2 (ng/L)	1.02 ± 1.33	0.91 ± 1.09	0.833
CHD	20 (14.49%)	24 (5.24%)	<0.001
Heart failure	4 (2.90%)	4 (0.87%)	0.088
DR	18 (13.04%)	3 (0.66%)	<0.001
DN	15 (10.87%)	3 (0.66%)	<0.001
dyslipidemia	94 (68.12%)	305 (66.59%)	0.818

Among the 6 laboratory indicators: DBP and VD2 were not statistically different between diabetic and non-diabetic patients(82.54 ± 9.42 VS 81.50 ± 10.81, p=0.091; 1.02 ± 1.33 VS 0.91 ± 1.09, p=0.833), and of the remaining 4 indicators: SBP were distinctively higher in the diabetic group than in the non-diabetic group (131.81 ± 18.11 VS 127.92 ± 17.83, p=0.004), while HDL-C, LDL-C were lower than in the non-diabetic group(1.21(0.99,1.44) VS 1.33(1.14,1.60),p<0.001; 2.75(2.18,3.33) VS 2.98(2.38,3.52), p=0.022), and CHOL were distributed at marginal levels that are not statistically significant(4.90(4.19, 5.69) VS 5.10(4.50, 5.95),p=0.047).

Regarding complications, our data show that CHD [20(14.49%) VS 24 (5.24%)], DR[18(13.04%) VS 3(0.66%)], and DN [15(10.87%) VS 3(0.66%)] are all more prevalent in diabetic patients(p<0.001), whereas the distribution of Heart failure [4(2.90%) VS 4(0.87%), p=0.088] and dyslipidemia [94(68.12%) VS 305(66.59%), p=0.818] in diabetic versus non-diabetic patients is not statistically significant. Some complications such as Heart failure having two boxes with data <5 when a chi-square test is performed, requiring a Fisher exact probability.

Logistic regression was performed between the two groups to adjust for confounding factors with statistically significant differences between the two groups. As shown in **Table 2**. It can be seen from the table that, after steply adjusting for confounding factors, the relationship between VD2 and T2DM is still not statistically significant. Even the crude OR does not change with increasing variables [OR: 1.08 (0.92,1.26)].

**Table 2:** Adjusted P value and odd ratio of VD2 and T2DM.

Model	crude OR(95%CI)	adj. OR(95%CI)	P (LR-test)
Model1	1.08 (0.92,1.26)	1.08 (0.92,1.26)	0.371
Model2	1.08 (0.92,1.26)	1.14 (0.88,1.47)	0.338
Model3	1.08 (0.92,1.26)	1.15 (0.88,1.49)	0.316

Model 1 adjusted for demographic characteristics (Age, BMI, smoking).

Model 2, adjusted for demographic characteristics (Age, BMI, smoking) and laboratory indicators (SBP, HDL-C, LDL-C, CHOL).

Model 3 adjusted for demographic characteristics (Age, BMI, smoking) and laboratory indicators (SBP, HDL-C, LDL-C, CHOL) and complications (coronary heart disease (CHD).

## Discussion

In this study, we used a metabolomics approach, and examined the association between T2DM and plasma VD2 in a cross-sectional study in China, aiming to identify new factors for T2DM, Finally, we found that there were no statistical association between plasma VD2 and T2DM. However, this result contradicts the previously generally accepted conclusion that VD deficiency can cause elevated blood glucose. Studies have shown that there are VD-related receptors in pancreatic β-cells, and when VD is insufficient, not only does it cause the closure of calcium channels and decrease insulin synthesis and secretion, but it also causes blocked phosphorylation of insulin receptor substrates and increases insulin resistance in the body; as a result, the body’s blood glucose rises (32–34).

After reviewing the relevant literature and team discussions, we found that the aforementioned literature refers to the relationship between VD and T2DM, and VD is a broad category in which no clear distinction is made. In fact, VD is found in the body in two main forms: VD2 and VD3 forms, but the two kinds of vitamins not physiologically active *in vivo*. They should be converted to 25-(OH)D in the liver by 25-hydroxylase and then further hydroxylated to 1,25-(OH)2D in the kidneys by 1α-hydroxylase before it becomes active and exerts its hypoglycaemic effect by binding to the VD receptor on the pancreas. There is a possibility it is true that there is no vital association between VD2 and diabetic patients and that the results we observed are true. A clinical trial showed that after 2 weeks of giving the same dose of VD3, VD2 and placebo to vitamin D deficient people, plasma 25(OH)D levels were significantly higher in the vitamin D intervention group, but those taking VD3 had significantly higher plasma 25(OH)D levels than the other two groups (35).

Of course VD2 can convert to VD, but its efficiency is far less than that of VD3 (36), which leads to that VD2 may have less impact on T2DM than VD3. In addition, in our study design, we did not review the patient’s diet and medication. It cannot be ruled out that the inpatients had a more balanced diet and were taking medications that included VD supplements. This is especially the case when several national and international authors have concluded that VD supplementation improves insulin resistance in T2MD patients (37–40). Moreover, in previous studies, VD2 was often used as an intervention in the form of supplements to the subjects, and the research type was usually cohort studies (41). Compared with cross-sectional studies without intervention factors, the actual relationship between VD2 and T2MD could be better detected. This means that in order to better explore the above relationships, we would do well to collect a balanced and comparable group of diabetic and non-diabetic patients and compare the differences between the two groups on the basis of controlled diet and medication.

There are a number of limitations to this study. Firstly, this study was a cross-sectional study and did not examine the causal relationship to the same extent as the cohort study (16). Considering that there are still relatively few studies on the relationship between VD2 levels and diabetes in China, the results of this paper are negative but may still provide some clues for further research. Secondly, given the limited time available for investigation, some lifestyle habits, such as dietary habits, may differ between diabetic and non-diabetic patients and should be further investigated. As a small metabolite, the level of VD2 in the blood reflects the environmental and nutritional status of the cells over a recent period of time. Therefore, in a sense, it is feasible to explore whether VD2 levels in blood can be used as a biomarker for T2DM. Finally, type 2 diabetic inpatients are severely ill and their usual therapeutic measures are likely to include VD supplements or to mask true differences (42). We gave preference to patients with a short duration of diabetes and hospitalisation for the study, but caution should be exercised when extrapolating this finding to the general population.

Overall, we did not find an statistical association between VD2 and T2DM in this study. Therefore, clinical caution should be exercised in giving VD2 supplementation to patients with T2DM unless other diseases requiring VD2 supplementation (e.g., rickets, osteoporosis) are present. I suggest that subsequent cohort studies with larger numbers of subjects should be conducted and interventions, such as oral VD2 supplements, should be administered to study subjects, to explore the association between the two.

## Data Availability Statement

The raw data supporting the conclusions of this article will be made available by the authors, without undue reservation.

## Ethics Statement

The studies involving human participants were reviewed and approved by The ethics Committee of Dalian R&amp;S Kangtai Medical Testing Laboratory Co. Written informed consent for participation was not required for this study in accordance with the national legislation and the institutional requirements.

## Author Contributions

BG designed the study. J-WQ analyzed the data and wrote the draft. BG, BH, SW, DS, JX and YC gave critical comments and contributed to the writing of this manuscript. All authors contributed to the article and approved the submitted version.

## Funding

This research was financially supported by the Development and Application of Clinical Mass Spectrometry Detection Technology for Important Small Molecule Metabolites (Project No. 2020JH2/10300116) and the Key R&D Program of Liaoning Province: platform name: Dalian Laboratory Medicine Mass Spectrometry Technology Innovation Center.

## Conflict of Interest

The authors declare that the research was conducted in the absence of any commercial or financial relationships that could be construed as a potential conflict of interest.

## Publisher’s Note

All claims expressed in this article are solely those of the authors and do not necessarily represent those of their affiliated organizations, or those of the publisher, the editors and the reviewers. Any product that may be evaluated in this article, or claim that may be made by its manufacturer, is not guaranteed or endorsed by the publisher.
